# Measuring molecular forces inside living cells using magnetic tweezers

**DOI:** 10.1007/s12551-025-01349-z

**Published:** 2025-08-07

**Authors:** Abhinav Kongari, Maxim Molodtsov

**Affiliations:** 1 The Francis Crick Institute, London, NW1 1AT UK; 2 Department of Physics and Astronomy, University College London, London, WC1E 6BT UK

**Keywords:** Magnetic tweezers, Force spectroscopy, Mechanical forces, Intracellular force measurement, Mechanobiology, Cell mechanics, Superparamagnetic particles

## Abstract

To change shape, move, grow and divide, cells employ various motor and non-motor proteins that convert chemical energy into the generation of mechanical force. Force spectroscopy tools that allow the measurement of these forces generated by individual molecules revolutionised our understanding of single-molecule mechanics over the past three decades. These techniques, however, remain largely confined to studies with purified components outside cells. A critical, unresolved challenge lies in deciphering how these force-generating and force-sensing molecules coordinate their activities inside living cells. In this review, we discuss advances in magnetic tweezers designed to measure and apply mechanical forces intracellularly. We highlight recent progress in magnetic tweezers that began to provide an understanding of how active mechanical forces drive rearrangements of biological structures. We also discuss challenges associated with applying forces locally and precisely. We identify two key areas that hold potential for the development of tools for direct mechanical manipulations of specific molecules inside living cells: (1) instrument design to generate and control magnetic gradients at the single-cell scale, and (2) development of magnetic biofunctionalised particles capable of targeting specific structures. The integration of these advances should enable unprecedented ability to manipulate intracellular forces, opening new avenues to study intracellular organisation, mechanotransduction pathways, cell division and migration. By addressing current limitations in specificity and resolution, next-generation magnetic tweezers may finally bridge the gap between single-molecule biophysics in vitro and cell-scale mechanobiology in living cells.

## Cellular structures are built by molecules that generate mechanical forces

Understanding mechanical forces that drive rearrangements of internal cellular structures is one of the long-standing questions in biology and it is at the heart of understanding biological self-organisation. All eukaryotic cells have unique shapes and complex internal organisation that are essential to their function. Understanding mechanisms that underlie assembly and rearrangement of intracellular structures such as mitotic spindles, chromosomes and complex cell shapes requires the ability to measure mechanical forces generated by molecules and molecular complexes that drive these changes.

**Fig. 1:**
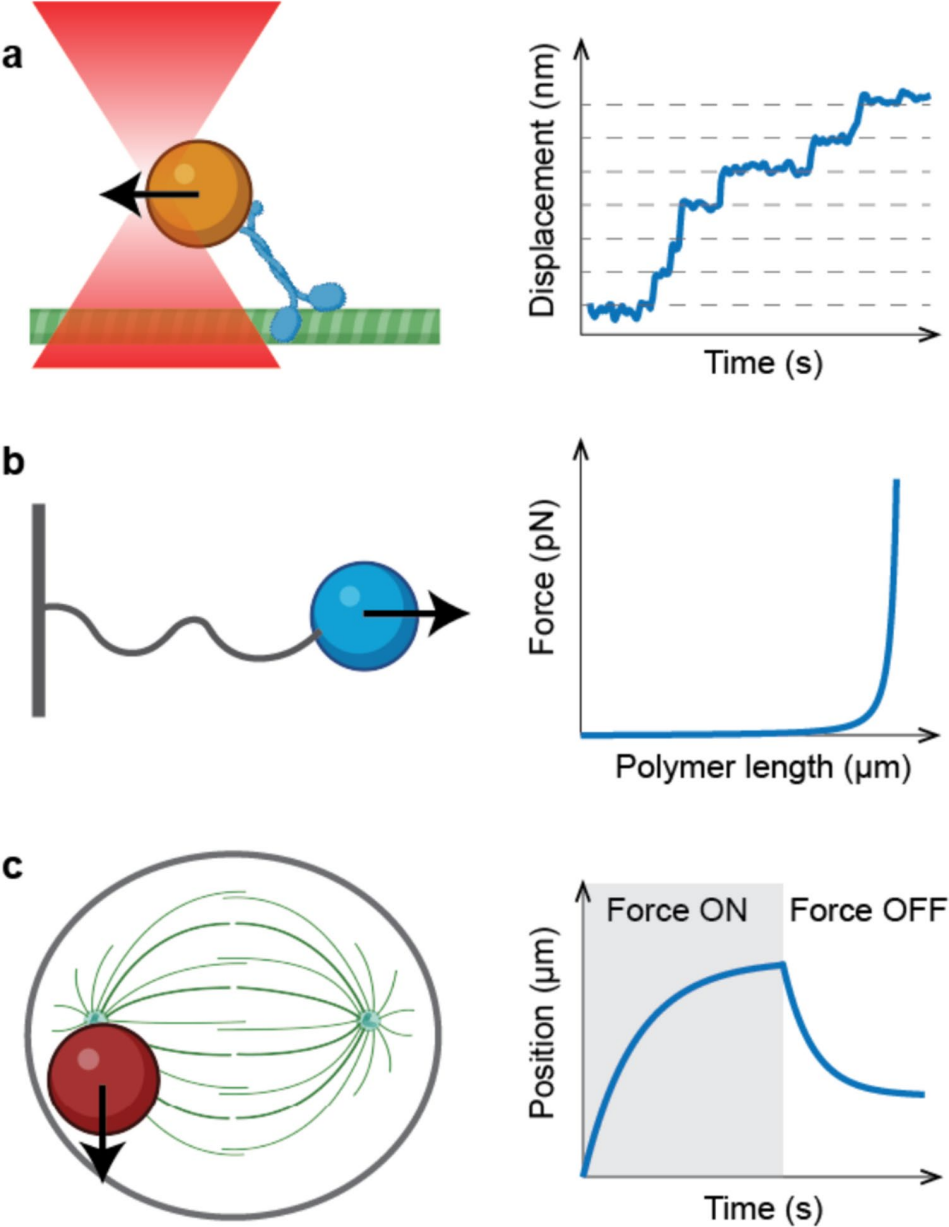
Forces acting on molecules, polymers and molecular systems. **a** Force applied to a single molecular motor allows one to identify its stepping motion and how the velocity of movement depends on the external force; **b** Force applied to a biological polymer allows extraction of its mechanical properties in the form of the force-extension curve; **c** Complex systems like the mitotic spindle exhibit a viscoelastic response when subjected to external forces: when the force displacing the spindle is applied, it takes time for it to adopt a new position (made partially with biorender.com)

While all molecules inside cells undergo random thermal motion, it is the generation or presence of external forces that biases their stochastic movement towards organisation into specific structures and intracellular architecture. To generate force, cells rely on specific molecules that convert chemical energy into mechanical work and unidirectional movement, and mechanisms used by many of these molecules are now beginning to be well understood. Progress has been made possible largely thanks to the development of force spectroscopy tools – a set of techniques that allow force to be applied to single molecules and measure their physical properties such as processivity, speed, elasticity and friction as functions of externally applied loads. Because molecules themselves are too small to be manipulated directly, all force spectroscopy tools rely on larger mechanical probes that typically are spherical particles that must be attached to molecules to exert force on them and measure their mechanical properties. Depending on the nature of the molecule or complex being investigated, the outcome of the experiment typically characterises how either movement or position of the molecule depends on the external force (Fig. 1).

Molecular motors are among the best-characterised molecules that generate mechanical force. These proteins convert the energy of ATP hydrolysis into directional movement. While the term molecular motors typically denotes cytoskeletal motors such as myosins, kinesins, and dyneins, this classification extends to diverse mechanochemical systems (Brenner et al. 2020; Can et al. 2019; Rief et al. 2000; Svoboda et al. 1993; Verhey et al. 2011; Wang et al. 1995).

Other molecular machines such as DNA/RNA polymerases exemplify linear track-following motors (Herbert et al. 2006; Hoekstra et al. 2017; Righini et al. 2018; Wuite et al. 2000), whereas rotary motion is exemplified by ATP synthase complexes (Itoh et al. 2004; Pilizota et al. 2007) and bacterial flagellar propulsion systems (Mandadapu et al. 2015; Nirody et al. 2017). Additional categories include polymerisation-driven motors such as growing actin and microtubules filaments, which generate protrusive forces through nucleotide-dependent assembly (Brouhard and Hunt 2005; Chu et al. 2024; Dogterom and Yurke 1997; Footer et al. 2007), as well as less conventional motors that reorganise DNA (Pobegalov et al. 2023; Ryu et al. 2022).

Molecular motors are not the only important components of the force generation. An important contribution is made by other molecules that sense mechanical force and change their shapes or kinetics in response to it. Thus, mechanical properties of all major polymers, intracellular filaments and DNA as well as viscoelastic properties of the cytoplasm and nucleoplasm are important for understanding basic cell functions and self-organisation (Xie et al. 2022).

The mechanisms underlying the actions of some molecular motors are now well characterised, and the mechanical properties of specific polymers such as DNA, actin, and microtubules are also well established. However, inside cells many motors, non-motor molecules and polymers work together and must coordinate their actions to produce accurate and reliable rearrangements of cell structures. How this coordination is achieved is much less well understood. Several studies indicate that the action of multiple motors may not be represented simply as a sum of their individual activities (Britto et al. 2016; Chu et al. 2024; Lansky et al. 2015; Molodtsov et al. 2016; Roostalu et al. 2011). Therefore, it is important to understand not only how the action of multiple molecules is coordinated, but also how they are integrated inside cells with activities of all other molecules and in the context of the polymers that they interact with.

This challenge is largely rather technical. To address this question, one must carry out measurements of forces and understand the emergent material properties of their collective action inside living cells. However, tools for measuring forces and manipulating molecules inside cells are limited. To design new tools to address this challenge, it is vital to know the magnitudes of forces to be measured. To aid with that, we have summarised the range of forces associated with a variety of intracellular systems in Table 1.

**Table 1:** Magnitudes of forces characterising various intracellular molecular systems

Generated force	System	Reference	Methods
1–6 pN	Single molecule of molecular motor kinesin	( Block et al. 1990; Carter and Cross 2006; Kojima et al. 1997 )	Optical trapping (recombinant proteins in vitro)
1–5 pN	Single molecule of molecular motor Dynein	( Anjur-Dietrich et al. 2024; Brenner et al. 2020; Ezber et al. 2020; Gennerich et al. 2007; Yildiz and Zhao 2023 )	Optical trapping (recombinant proteins in vitro)
3–5 pN	Single microtubule filament polymerisation	( Dogterom and Yurke 1997; Kent and Lele 2017; Schek et al. 2007 )	Optical trapping (recombinant proteins in vitro)
30–65 pN	Depolymerisation of a single microtubule	( Driver et al. 2017; Grishchuk et al. 2005; Volkov et al. 2013 )	Optical trapping (recombinant proteins in vitro)
~ 1.3 pN	Polymerisation of single actin filament	( Footer et al. 2007 )	Optical trapping (recombinant proteins in vitro)
20–60 pN	Whole spindle displacement (*C. elegans*)	( Garzon-Coral et al. 2016 )	Magnetic trapping inside embryos
0.1 - 1 pN	DNA locus displacement (human cell nucleus)	( Keizer et al. 2022 )	Magnetic trapping inside cultured cells

## Measuring forces inside living cells using force spectroscopy

Improving force spectroscopy to measure forces inside living cells has been widely accepted as the next big challenge in the field. The two techniques that can be used for this purpose are optical and magnetic tweezers because they allow remote operation of probes—by laser light or magnetic field, respectively. If probes can be delivered inside cells without significant damage to the cellular function, this allows forces to be exerted on them via light or magnetic field without direct contact with the measurement device, thus enabling measurements in live cells.

Optical trapping uses tightly focused laser beam transmitted through high-numerical-aperture microscope objectives to exert precise, piconewton-scale forces on dielectric particles such as polymer microspheres. This approach is based on momentum transfer during light refraction at the interfaces between materials with differing refractive indices and enables nanometre-resolution manipulation of functionalised beads (Block et al. 1990; Davenport et al. 2000; Finer et al. 1994; Kuo and Sheetz 1993). When coupled with biological molecules—including nucleic acids, proteins, or molecular motors—these systems have revolutionised single-molecule biophysics by permitting direct observation of mechanochemical processes.

Optical trapping remains the gold standard for quantifying forces generated by molecular motors, polymers and cytoskeleton filaments in cell-free reconstitution experiments. However, accurate force calibration relies critically on the refractive index contrast between trapped beads and their surrounding medium. This is readily achieved in controlled buffer systems but is more challenging inside the crowded environment of living cells, where light scattering from membranes, organelles, and cytoplasmic constituents can distort the calibration and special measures need to be taken to account for these effects. Some progress has been made in overcoming this (Blehm et al. 2013; Hendricks et al. 2012), but another problem is that organelles and vacuoles are attracted towards higher intensities of the beam making it impossible to trap one selected specific object. This was exploited to measure the mechanics of intracellular junctions by exerting force on them directly without using any additional probes (Bambardekar et al. 2015) but presents a significant challenge for making accurate measurements otherwise. In addition, tens of piconewtons or larger forces that are typically required to cause detectable deformations in living cells also require large power densities for trapping (typically 100–1000 mW/μm^2^). Local heating and photodamage by such laser intensity frequently risk confounding experimental outcomes (Neuman et al. 1999; Peterman et al. 2003).

Magnetic tweezers seem to avoid both disadvantages. Cells are typically non-magnetic and therefore forces applied to superparamagnetic particles inside cells are not affected by their surroundings. Although heating of cells may be an important consideration in magnetic tweezers too, there are current designs that avoid this problem.

## Problems considered here

Mechanical forces are an integral aspect of biology across all scales from atoms to organisms. Here, we focus only on how to quantify forces at the mesoscale, i.e., the scale at which macromolecules assemble into intracellular structures inside cells. In other words, we are discussing how to map forces that position macromolecules and organelles such as nuclei, mitochondria, cytoskeleton filaments, and chromosomes.

We will limit our consideration to magnetic tweezers, and we will also limit ourselves to magnetic tweezers used specifically for intracellular cell application. Other types of magnetic tweezers have been instrumental for understanding mechanics of macromolecules in vitro and outside the cells. For those, we refer readers to excellent reviews elsewhere (Bell and Molloy 2023; Berghuis et al. 2016; De Vlaminck and Dekker 2012; Dulin 2024; Kriegel et al. 2017; Lipfert et al. 2010; Tapia-Rojo 2025).

## Magnetic tweezers operating principle

**Fig. 2:**
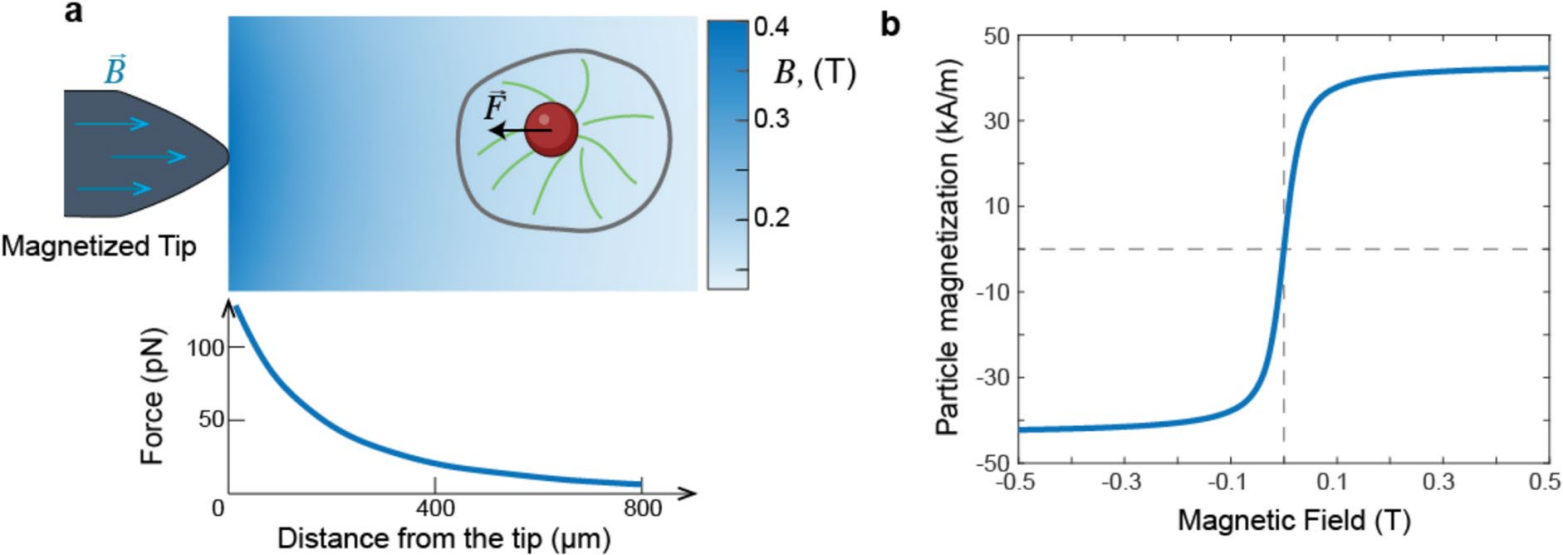
Magnetic tweezers operating principle. **a** Sharp magnetized tip generates a steep gradient of magnetic field ($B$), which quickly drops with the distance from the tip. The force applied to a magnetic particle depends on the distance from the tip. Force data is for 0.5 mm diameter tip and 1 micron-sized bead from (Bijamov et al. 2010); **b** Magnetization of 1 µm MyOne Dynabead as a function of the external magnetic field using data from (Lipfert et al. 2009a, b)

The general setup to apply forces inside cells by magnetic tweezers is shown in Fig. 2a. It consists of a magnetic probe with the diameter sized generally between 1 and 2.8 µm, delivered inside a cell and an external magnetic device that generates a magnetic field, which applies the force to the magnetic particle. The size of the magnetic particle determines the amount of the magnetic material inside it and therefore the maximum force that it can exert. Thus, the size of the particle is an important consideration for applying magnetic tweezers in live cells—larger particles provide more force but are more challenging to deliver and control inside cells. Because this trade-off is important for choosing particles, we will first consider how magnetic tweezers generate force and how force depends on the properties of the particle and the magnet.

The force acting on a magnetic particle in a magnetic field is calculated by:

1 $$F=\left(m\cdot\nabla \right)B$$

where $\text{m}$ is the magnetic moment of the particle, and $\text{B}$ is the field generated by the magnet at the particle location.

Magnetic tweezers use superparamagnetic particles as probes. Superparamagnetic particles are particles that are not magnetic in the absence of an external field but acquire an induced magnetic moment when placed in it. (Ferromagnetic particles, which are constantly magnetized would be impossible to use as they would stick to one another like tiny magnets). For superparamagnetic particles induced magnetisation increases with the increasing external field generated by the magnetic tweezers. In larger fields the relationship becomes nonlinear, and magnetization saturates (Fig. 2b). For particles made of iron oxide, which is one of the most magnetic materials and frequently used for particles in magnetic tweezers, the magnetization saturation field is below 0.5 T (Lipfert et al. 2009a, b; Lisse et al. 2017). This value is easily achieved in most setups and, therefore, increasing the field further does not increase the particle magnetization. Thus, the absolute value of the force applied to a magnetic particle is calculated by:

2 $$F={m}_{sat}\cdot \rho V\cdot \nabla B$$

In the above equation, ${m}_{sat}$ is the saturation magnetisation, which is the parameter characterising the material from which the particle is made (i.e., iron oxide), $\rho V$ – is the density times the volume of the particle, $\nabla B$ is the gradient of the magnetic field. This shows that the only two parameters that significantly affect the force applied to the particle, which can be experimentally controlled, are the volume of the particle (for composite particles, this is the amount of magnetic material in it) and the gradient of the external field. This also shows that magnetic force scales with the volume of a particle. Thus, if magnetic tweezers are capable of exerting 50 pN on a 1 micron-sized particle, in the exact same conditions, they should only exert 0.05 pN on particles 0.1 micron in size, making the usage of small particles very challenging.

Another part of the magnetic tweezers is the device that generates the magnetic field. As follows from the consideration above, when the size of the particle is limited, increasing the magnetic gradient is the only way to increase the force acting on the particle. Therefore, all magnetic tweezers that apply forces in live cells aim to generate steep magnetic gradients to maximise force. The simplest way to generate steep gradient of the field is to manufacture a sharp edge or a corner. At an edge, the normal direction of the field must change abruptly from one surface to another and therefore produce a high gradient. In practice, this can be achieved by using the tip of a microneedle as in Fig. 2a, which is either permanently magnetised, or whose magnetisation is controlled by an electromagnetic coil wrapped around the needle that acts as a core of the electromagnet.

The strength of the gradient that can be generated by the magnetic tip depends on the material of the core, parameters of the coil, but also on the geometry of the core at its tip, where the gradient is the steepest (Fig. 2a). Sharper tips generally generate steeper magnetic gradients, which have the potential to apply stronger force for the same size particle (Bijamov et al. 2010; Matthews et al. 2004). However, for very sharp tips the size of the particle and the distance between the particle and the tip will affect the maximum achievable force. In principle, fine details of the tip geometry that affect forces generated by magnetic needles could be matched to the requirements of specific experiments and optimised for the use of the specific particles and cellular systems. For example, sharper tips with stronger gradients could be used in experiments with smaller cells, while larger tips with broader gradients could be used in larger cells and embryos.

Before magnetic tweezers can be used for measurements, they need to be calibrated. Calibration is the procedure of establishing an unambiguous relationship between the position of the bead with respect to the magnetic field and the force applied to it by the magnet. Once this relationship is established, the force in real experiments can be inferred by measuring the position of the bead relative to the tip of the magnet. Calibration is typically done by taking advantage of the fact that magnetic forces are independent of the medium they are in. Sparsely populated beads are suspended in a viscous medium with known viscosity such as calibrated oil or glycerol and the magnetic tweezers tip is positioned at a specific distance from the chosen bead. Once the current is turned on, the bead starts moving towards the tip at a velocity at which the force exerted by the magnet is balanced out by the Stokes drag acting on the bead. The force is calculated by the Stokes viscous drag force formula giving the desired relationship between the bead position with respect to the tip and the applied force (Kah et al. 2020; Kollmannsberger and Fabry 2007; Moghram et al. 2021). This relationship is later used in the experiment to determine the unknown force from the distance between the bead and the tip.

## Existing approaches to generate magnetic gradients for intracellular manipulation

**Fig. 3:**
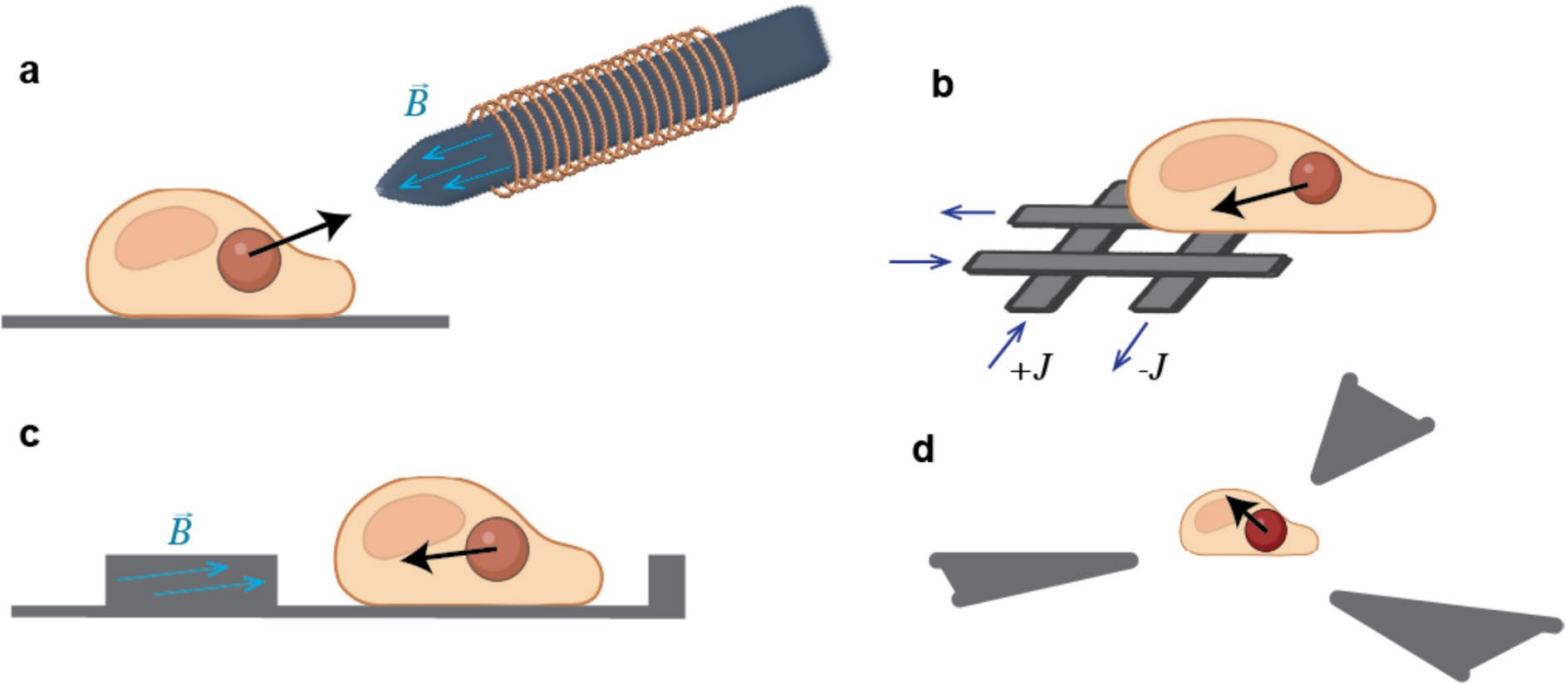
Different approaches to generating magnetic field gradients to exert force on particles inside cells.** a** Microtip magnetic tweezers generate gradient of magnetic field using sharp highly magnetized tip attached to a micromanipulator that allows its 3D positioning; **b** Microwire matrix generates localised gradient of magnetic field by running opposing currents ($J$) in closely spaced microwires; **c** Highly magnetized micropatterned micropillars generate magnetic gradients at their boundaries; **d** By using multiple poles whose magnetization is controlled by independent currents force applied to the particle can be fully controlled in 3D. Black arrows show direction of the force applied to the particle

A sharp magnetic tip is a convenient way to generate a magnetic field with steep gradients. When attached to a 3D micromanipulator or a 3D piezo controller, the tip can be precisely positioned with respect to a cell on a regular Petri dish (Fig. 3a). The system can be easily set up on a conventional inverted microscope that is available in many biological laboratories. However, some limitations include a lack of control with respect to the direction of the applied force, which is always directed towards the tip. The tip also needs to be positioned within microns or tens of microns from the cell, which means it must be inserted inside the media or the buffer. That may be limiting for some applications. Alternative approaches for generating sharp magnetic gradients have been developed, with some specifically aimed at overcoming these limitations and will be discussed below.

### Microwire matrices

Localised magnetic fields can be produced by passing electric currents through microwires lithographically patterned on a substrate and covered with an insulator layer on which cells can be cultured (Lee et al. 2004; Shevkoplyas et al. 2007). In the simplest configuration, two perpendicular pairs of wires in which the current runs in the opposite directions generate localised magnetic fields with a very sharp gradient determined by the distance between microwires (Fig. 3b). Multiphysics simulations revealed that the maximum force applied to magnetic particles is reached when the distance between the wires is in the range of 10–20 microns (Kongari 2024). Smaller distances require smaller wires, which increases the resistance, while larger distances decrease the amplitude of the gradient. However, even at the optimal distance, the maximum force that microwires can exert on a 1-micron size particle is less than 2 pN (Kongari 2024). This also requires currents over 1 A, which when run through wires on the scale of microns generate significant heat even in the presence of heat transfer modules and would be incompatible with live cell experiments. Other disadvantages include poor flexibility as the position of the gradient is determined by the existing wire pattern that cannot be changed, and a magnetic field that quickly decays with increasing distance from the surface. Thus, while microwire matrices offer significant potential for manipulating microparticles in controlled environments, their limitations have so far prevented their use for live-cell applications.

### Magnetic micropillars

Strong magnetic forces can be applied to magnetic particles by magnetic micropillars fabricated on a planar substrate (Fig. 3c) (Bidan et al. 2018). Micropillars are typically made from highly magnetic alloy, which in the presence of an external magnetic field is magnetized similarly to the core of magnetic tips (le Digabel et al. 2011). Therefore, magnetic pillars can create strong gradients comparable to those generated by microtips and microneedles with an additional benefit that thousands of micropillars can be magnetized at once allowing for highly parallel application (Tseng and Di Carlo 2014). These have been used to direct cell migration and induce neurite extension in response to mechanical stimuli without compromising cell viability (Cardoso et al. 2018; Nagayama et al. 2018; Vining and Mooney 2017). They have also been recently used to study the mechanics of chromatin (Keizer et al. 2022). The major limitation of the magnetic pillars is that their positions are fixed with respect to cells and therefore the force exerted on particles inside cells cannot be controlled other than being switched on and off. Thus, although this technique allows for hugely parallel force applications, it may be less suitable where spatiotemporal control over the applied forces is required.

### Multipole magnetic tweezers

Multipole tweezers have been developed to address the main limitation of single-tip instruments that can only apply pulling force in one direction and cannot form stable traps as, for example, in optical tweezers. They use several magnetic tips positioned around the sample and an integrated feedback loop to generate a stable magnetic trap that can apply force in any direction. Since all tips need to be positioned within tens of microns from the cell to generate sufficient force, modern multipole magnetic tweezers combine a multiplexed electronics setup with microfabrication techniques (Fig. 3d). The poles are typically electroplated onto glass substrates in predefined patterns. Researchers have developed systems with up to six magnetic poles arranged in specific geometries to enable full three-dimensional manipulation of magnetic probes (Fisher et al. 2005; Gosse and Croquette 2002). Others further demonstrated the capability of multipole devices to control single magnetic beads in three dimensions (Chiou et al. 2006; Zhang et al. 2019), along with force feedback that can provide the functionality of a position clamp (Kanger et al. 2008). Advanced multipolar magnetic systems can apply over a hundred of piconewtons force to 2.8µm sized particles for periods longer than 30 min, allowing for extended observation of cellular responses to mechanical stimuli (Chen et al. 2015; de Vries et al. 2005; Wang et al. 2019a, b). However, for these high force experiments, single cells need to be positioned within close distance to the poles in the centre of the multipole device. This makes high force multipole magnetic tweezers a very low throughput and difficult to use instrument, which is poorly suited for a general biology laboratory. Poles can be positioned further away for easier operation, but that compromises the amount of force that can be generated by the system.

## Applications of magnetic tweezers inside living cells

Magnetic tweezers were originally used to study the viscosity of protoplasts along with further uses in understanding the rheology within echinoderm eggs (Heilbronn 1922). Dr Honor Fell further suggested that tiny magnetic beads could be engulfed by live cells and manipulated using an external magnetic field. Crick and Hughes took this idea forward and measured the viscoelasticity of the cytoplasm (Crick and Hughes 1950). Thus, the use of magnetic tweezers to manipulate cells dates back to early twentieth century. However, applying targeted and strong forces to specific intracellular structures has remained a challenge. Although some very advanced magnetic tweezers instruments have been recently developed, one of the most important design considerations of magnetic tweezers is the ease to use in a general biological laboratory. Thus, most major discoveries made with the help of magnetic tweezers applied inside living cells came from using simple assays employing a single sharp tip capable of generating strong force. We discuss these examples below.

One area where magnetic tweezers were successfully applied intracellularly is understanding the positioning of the mitotic spindle—a molecular machine assembled from microtubule polymers whose role is to segregate sister chromatids during cell division. Accurate assembly and positioning of the mitotic spindle are vital for cells as errors lead to cell death and aneuploidy. To avoid that, cells use multiple mechanisms to ensure that spindles are accurately and reliably assembled and positioned. By injecting 1-micron sized magnetic particles into *C. elegans* gonads, Garzon-Coral was able to isolate embryos in which beads were located close to the spindle during the first embryonic division. They then applied a calibrated force to the bead which displaced the spindle and allowed measurement of the forces that keep the spindle centred (Garzon-Coral et al. 2016). Similar experiments were recently made in human cells that could passively uptake magnetic particles. In mitotic cells where one particle randomly ended up positioned close to the spindle pole, the force rotating the spindle was applied by a magnetic tip through non-specific interaction between the particle and the dense array of spindle microtubules (Anjur-Dietrich et al. 2024). This study revealed that spindle orientation is determined by pulling forces on astral microtubules generated by single dynein motors localised at the cell cortex. A similar setup allowed manipulation of the spindle position and orientation by magnetic tweezers using 1 µm sized magnetic particles in developing sea urchin embryos (Xie et al. 2022). This allowed quantification of the forces and torques needed to move or rotate spindles as well as to quantify hydrodynamic interactions of structures in the embryo cytoplasm (Najafi et al. 2023).

Another area where magnetic tweezers applied intracellularly yielded interesting results is the characterisation of nuclear mechanics and how it responds to mechanical stimuli (Wang et al. 2019a, b). These measurements revealed that the nucleus exhibits significantly higher stiffness along its major axis compared to its minor axis, a property likely attributed to aligned actin filaments. This may contribute to how mechanotransduction is controlled on the nuclear envelope as shown in a study where magnetic tweezers were used to apply force to nuclei isolated from cells (Guilluy et al. 2014).

Magnetic tweezers used intracellularly with particles freely diffusing in the cytoplasm allowed for several interesting discoveries. For example, this was used for the generation of controlled signalling gradients inside cells by attaching particles to intracellular molecules that can be redistributed using magnetic forces (Etoc et al. 2015; Lisse et al. 2017; Neusch et al. 2024). Manipulating freely diffusing magnetic particles in cells is a powerful approach for interrogating cytoplasmic mechanics (Bausch et al. 1999), which allows measurement of local viscoelastic moduli, active forces and shear viscosity of the cytoplasm inside living cells (Berret 2016; Feneberg et al. 2001; Wilhelm et al. 2003). By using magnetic tweezers to move passive components through the cytoplasm, it was shown that cytoplasmic crowding, viscoelasticity and hydrodynamic interactions may be important contributors to the generation of forces that position organelles at the micrometre scale (Najafi et al. 2023; Xie et al. 2022).

Recently, passivated ferrofluids emerged as an alternative to magnetic nanoparticles because they can be more readily injected inside cells as compared to large magnetic probes. A small amount of passivated ferrofluid injected inside cells was used to interrogate the mechanics of the cytoskeleton (Orii and Tanimoto 2025) and infer mechanical differences between the cytoplasm and actin cortex in Drosophila embryos (Doubrovinski et al. 2017).

Another application where the use of magnetic particles intracellularly promises great impact is understanding 3D organisation of chromatin. An earlier study manipulated passivated particles in live cell nuclei using multipolar tweezers (Kanger et al. 2008). However, in all applications mentioned above, magnetic particles were not attached specifically to any target inside cells and exerted their forces non-specifically. Recently, in breakthrough experiments, specific attachment of many small magnetic particles to chromatin inside nuclei allowed for the quantification of the physical properties of chromatin directly in live cells (Keizer et al. 2022). This opens up new ways to manipulate specific molecules in live cells and will allow us to understand how physical interactions between them enable cells to build complex internal architectures.

## Future directions

Magnetic tweezers for intracellular measurements keep improving. Recent advances allow actively controlling magnetization of particles (Kah et al. 2020; Moghram et al. 2021), integration with temperature and CO_2_ control (Aermes et al. 2020) and combination with other imaging and force-spectroscopy techniques (Shepherd et al. 2024).

However, application of targeted forces to specific molecules in living cells remains challenging. Except for one study (Keizer et al. 2022) and a notable exception (Tanimoto et al. 2018), where minus-end directed motors were non-specifically recruited to streptavidin coated particles, which targeted them to the microtubule organising centres, in all other studies the force was applied without specifically attaching particles to specific targets inside cells. To enable force application on specific intracellular molecules in the future, magnetic probes will need to be targeted and bound to them with high specificity.

Specific attachment could be achieved by using interaction between GFP nanobody at the target protein and GFP at the magnetic particles as in (Keizer et al. 2022) as well as using other chemical crosslinking such as FRB-FKBP interaction, SNAP, HALO and SpyTag systems (Banaszynski et al. 2005; Hussain et al. 2013; Ma et al. 2018; Wang et al. 2019a, b). However, while particles need to interact specifically with the target, they should also be sufficiently passivated to prevent their interaction with everything else. Given the highly crowded intracellular environment, which includes physically diverse structures such as cytoskeleton, ER and others, finding the appropriate magnetic particle functionalization and surface chemistry represents formidable challenge. One of the possible directions to explore could be using the genetically encoded magnetic particles (Clarke and Royle 2018; Li et al. 2019), but whether they can be engineered to generate sufficient force is unclear.

The size of particles also presents a challenge. Injection of sufficiently large particles to exert tens of piconewtons of force inside cells requires large pipets and is damaging to cells. Passive uptake is possible in some cases, but it engulfs beads in membranes, which significantly complicates functionalization of beads for targeting to specific structures. Another limitation that comes with the large size of particles is that if the force needs to be applied to structures smaller than the mitotic spindle (e.g. mitochondria, specific gene loci, individual microtubules, etc.), nonspecific interactions between the bead and other intracellular components complicate the interpretation of the measurements. Therefore, one of the interesting future directions should be to explore how magnetic gradients can be maximised to manipulate smaller beads with sufficient force that could target structures smaller than mitotic spindles.

**Fig. 4:**
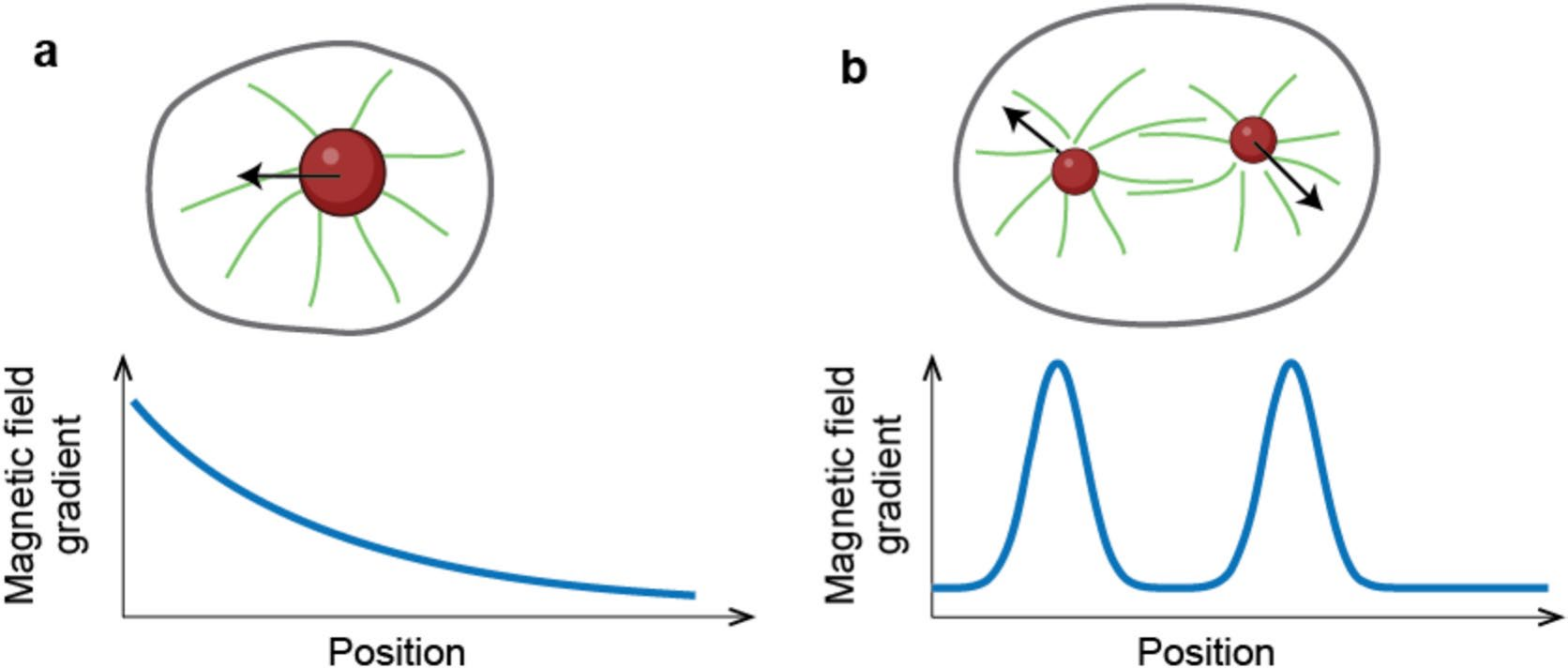
Future directions in magnetic tweezers. **a** Currently, large single bead can be manipulated inside cell by placing it in the magnetic field gradient shown in the bottom; **b** Independent manipulations of several beads inside cells are required for applying more diverse set of interrogations like rotation or stretching. This may potentially be achieved by generating more localised steeper gradients

The other limitation of magnetic tweezers is that they can only control independently the position of one bead inside a cell. This imposes limitations on the types of manipulations that can be carried out by the system. For example, rotation or stretching requires application of forces from two sides, and, therefore, the ability to independently manipulate two beads in the same cell, which is not possible at the moment (Fig. 4). If multiple beads can be delivered inside cells and attached to specific positions, magnetic fields generated by magnetic tweezers would act similarly on them. To manipulate multiple beads independently, one would have to generate very sharp non-overlapping magnetic gradients at the subcellular scale. Generating such gradients would be an interesting and important direction for future work.

Decreasing the size of particles should in future allow improvements on another single most challenging limitation of the magnetic tweezers – the fact that particles first need to be delivered inside cells. In cases where surface chemistry of magnetic nanoparticles is used to target them to specific structures inside cells the only method available for delivery so far in microinjection. Decreasing the size of particles should allow using thinner pipets and make injections much easier for cells to tolerate. Other ways of delivering particles inside cells should also be explored.

Finally, throughput and the ease of use are important considerations when choosing an intracellular force application strategy that can be used in a biological laboratory. Combining the throughput of the micropillar approach with the precision and flexibility of the microtip measurements would reveal unprecedented details of intracellular mechanics.

## Data Availability

No datasets were generated or analysed during the current study.
